# How Effective Is Help on the Doorstep? A Longitudinal Evaluation of Community-Based Organisation Support

**DOI:** 10.1371/journal.pone.0151305

**Published:** 2016-03-11

**Authors:** Lorraine Sherr, Alexa R. Yakubovich, Sarah Skeen, Lucie D. Cluver, Imca S. Hensels, Ana Macedo, Mark Tomlinson

**Affiliations:** 1 Department of Infection & Population Health, University College London, London, United Kingdom; 2 Centre for Evidence-Based Intervention, Department of Social Policy & Social Intervention, University of Oxford, Oxford, United Kingdom; 3 Department of Psychiatry and Mental Health, University of Cape Town, Cape Town, South Africa; 4 Department of Psychology, Stellenbosch University, Stellenbosch, South Africa; University of Stellenbosch, SOUTH AFRICA

## Abstract

Community-based responses have a lengthy history. The ravages of HIV on family functioning has included a widespread community response. Although much funding has been invested in front line community-based organisations (CBO), there was no equal investment in evaluations. This study was set up to compare children aged 9–13 years old, randomly sampled from two South African provinces, who had not received CBO support over time (YC) with a group of similarly aged children who were CBO attenders (CCC). YC baseline refusal rate was 2.5% and retention rate was 97%. CCC baseline refusal rate was 0.7% and retention rate was 86.5%. 1848 children were included—446 CBO attenders compared to 1402 9–13 year olds drawn from a random sample of high-HIV prevalence areas. Data were gathered at baseline and 12–15 months follow-up. Standardised measures recorded demographics, violence and abuse, mental health, social and educational factors. Multivariate regression analyses revealed that children attending CBOs had lower odds of experiencing weekly domestic conflict between adults in their home (OR 0.17; 95% CI 0.09, 0.32), domestic violence (OR 0.22; 95% CI 0.08, 0.62), or abuse (OR 0.11; 95% CI 0.05, 0.25) at follow-up compared to participants without CBO contact. CBO attenders had lower odds of suicidal ideation (OR 0.41; 95% CI 0.18, 0.91), fewer depressive symptoms (B = -0.40; 95% CI -0.62, -0.17), less perceived stigma (B = -0.37; 95% CI -0.57, -0.18), fewer peer problems (B = -1.08; 95% CI -1.29, -0.86) and fewer conduct problems (B = -0.77; 95% CI -0.95, -0.60) at follow-up. In addition, CBO contact was associated with more prosocial behaviours at follow-up (B = 1.40; 95% CI 1.13, 1.67). No associations were observed between CBO contact and parental praise or post-traumatic symptoms. These results suggest that CBO exposure is associated with behavioural and mental health benefits for children over time. More severe psychopathology was not affected by attendance and may need more specialised input.

## Introduction

In the era of HIV infection, children are increasingly vulnerable. The high death rate and burden of the illness in those infected–especially in resource-poor settings–has impacted considerably on family life and functioning [1]. Since the roll-out of antiretroviral treatment, mortality and morbidity has improved, but the impact of the disease on children remains high [2]. The legacy of HIV-related mortality cannot be reversed, and many children have lost their parents and caregivers to the disease, or live with families where adults or children are living with HIV [3]. In high endemic countries all village children may be touched in some way by the ravages of HIV. These environmental challenges pose a risk for children and their capacity to reach their full developmental potential. They face multiple adverse events compounded by poverty, frequent family movement, separation and stigma. As a result, global agencies focused on targeting orphans and vulnerable children, and a considerable injection of resources has been mobilised at the grassroots level to respond to the needs of such deprived families [4]. Community-based organisations have been used as a response to various health and social needs over time [5]. This has been with mixed effects. Often it has been a low-cost option in resource-limited or expertise-constrained environments [6]. The growth of community-based organisations (CBOs) to meet the needs of families in the HIV epidemic has been well-documented [7]. There is emerging evidence of the role of such provision for treatment and adherence interventions. However, the evidence base for the impact of such provision on child outcomes has been slow to emerge [8]. An early review failed to identify a single study that met inclusion criteria for evaluation of such interventions to improve the psychosocial well-being of children [9].

There are considerable challenges to adequately evaluating community-based programmes. These challenges relate to the difficulty in weaving high-quality research designs around already-established provision, the lack of research skill and funding for evaluation at the front line with competing high demands, and the overuse of descriptive evaluations which cannot provide insight into causal relationships [10]. Randomised controlled trials are considered to be the gold standard of programme evaluation and provide the strongest evidence for a programme’s effectiveness [11; 12]. However, they are often not suitable for evaluating community programmes, in part because of the high costs and drain on other programme resources, but also because of small numbers, fear of funding loss, and lack of skill and opportunity. When long-term care is being provided the number of new children receiving services in already-established organisations may be quite small, therefore impacting on the feasibility of generation sufficient sample size. In contexts where programs are operating, it may be unethical or unacceptable to withhold interventions from a control group [13]. Finally, there may be reluctance at times for organisations to be involved in research as it may represent a conflict of interest, a threat at worst or an inconvenience at best. Evaluations of community-based interventions set up as a research study may not reflect field reality and findings may not generalise.

CBOs vary considerably in their programme content and methods of delivery. Yet in the current phase of evidence-based programming, the need for guidance and insight into efficacy is acute [14]. Core components of community programmes may need to be distilled for evaluation purposes rather than focussing on micro-components of service delivery [15]. As funding resources dwindle, investment in efficacious interventions is preferable and competition for resources may leave the unevaluated as a poor option for funding. Although CBOs have different structures and different provisions, they have much in common. They reach the most vulnerable groups [16], they are located within the affected communities, they are located at the front line with highly vulnerable families, they are accessible to the most difficult to reach, they are focused on the family for the most part and they utilise a number of common provisions.

In the face of the need for evidence in this area, and a lack of studies from which we can draw conclusions, creative solutions, such as merging existing data from existing studies, may provide some preliminary evidence for the impact of CBO programming on children. This study utilised data drawn from South African samples in two different longitudinal studies with intentionally-shared information collected. The first study was to examine the impact of community-based programming to improve child psychosocial well-being and the second was a household survey of children. The household survey had detailed questions on service use and was thus able to generate a comparison group with no CBO contact over time. In doing so, the study compared child outcomes for a sample of children attending CBOs over a period of 12–15 months against those of a sample of children who had received no CBO interventions, drawn from a large-scale random sample. This longitudinal data analysis is set up to examine change over time in order to explore the overall effects of CBO enrolment on a range of child outcomes.

## Method

### Participants and procedure

Data were drawn from two longitudinal studies, the Child Community Care study (CCC) and the Young Carers study (YC). Data on children who attend CBO programmes were drawn from CCC, and data on children who did not were drawn from YC.

#### Child Community Care study

The Child Community Care study is a multinational study of children affected by HIV/AIDS between the ages of 4 and 13 years, receiving services from CBOs in two Southern African countries, South Africa and Malawi. This analysis was confined to the South African data only. In order to select CBOs to participate, 11 funding partners provided researchers with the names of all CBOs that they supported that directly provided services to children. From the list of 588 CBOs, a random sample of 28 CBOs (24 in South Africa and four in Malawi) was generated, stratified by funder and geographical region. From these, consecutive children (approximately 35 from each CBO) were enrolled in the study (2011–2012). Inclusion rate was high (above 99%) and a sample of 989 children was enrolled at baseline. At follow-up (12–15 months later) there was an 86% retention rate. Participants completed a face-to-face interview with a trained data collector using a mobile phone. Questionnaires were translated and back-translated into Zulu and Xhosa.

#### Young Carers study

For the Young Carers study, participants were randomly selected from two urban and two rural health districts with over 30% antenatal HIV prevalence in two South African provinces. Sampling involved randomly selecting census enumeration areas from the four health districts, visiting every household in the selected areas, and randomly selecting one child from every household with a resident aged 9–18. Refusal rate at baseline was less than 2.5%. Participants were interviewed at baseline (2009–2010) and one-year follow-up (2011–2012) with 96.8% retention. Participants completed a 60-minute face-to-face interview in the language of their choice. Interviewers were trained and experienced in working with vulnerable children and questionnaires were translated and back-translated in Xhosa, Zulu, Sotho, and Shangaan. Questionnaires including detailed measures of CBO provision were used to establish a subgroup of children from the YC study who had no exposure at either baseline or follow-up to any form of CBO service.

The sample for the analyses reported here was extracted from the larger databases of the two studies, based on the overlapping age range of 9–13 years. Only South African data was used. For the Child Community Care study, there were 446 children eligible for inclusion. For the Young Carers study there were 1402 children eligible for inclusion. Thus the total sample for the study was 1,848 South African children aged 9–13 years.

#### Ethical procedures

For the CCC study, ethical approval was granted by Stellenbosch University and University College London ethics committees and by the funding agencies that supported the CBOs. For the YC study, ethics approval was received from the Universities of Oxford, Cape Town, and KwaZulu-Natal and all provincial Health and Education Departments: Oxford University Central Research Ethics Committee (CUREC), University of Cape Town Health Sciences Research Ethics Committee, University of KwaZulu Natal Research Ethics Committee, Western Cape Provincial Department of Health, Western Cape Provincial Department of Education, Mpumalanga Provincial Department of Health, Mpumalanga Provincial Department of Education. In both studies, caregivers and child participants provided voluntary informed consent. In the CCC study written consent from the caregivers and verbal assent from the children was obtained. In the YC study written consent was obtained from caregivers and children. Participants received either refreshments or small care packages and certificates for their participation in both studies. Confidentiality was maintained in both studies, except when participants were at risk of significant harm or requested assistance. In the CCC study, referrals were made to the partner community organisations or local services for support, and in the YC study, referrals were made to the local social and health services with follow-up support.

### Measures

The studies had a number of measures in common which were used in the analysis.

#### Socio-demographics

Age and gender were measured using national census items in both studies [17]. (In)formal housing was measured by having participants indicate in which of different types of houses they lived (i.e. a house/flat, a shack, or on the street). Orphanhood was defined in accordance with UNAIDS as the loss of one or both biological parents in both studies [18]. Household size and employment was measured in both studies by having participants count how many people live in their home and the employment status of each. In YC, school enrolment was measured using items developed in collaboration with the national Department of Basic Education and corroborated using school registers. In CCC, school enrolment was determined on the basis of child and caregiver report. In both studies, community violence was measured using two child-report items of the Child Exposure to Community Violence Checklist [19], having (a) seen someone being attacked and (b) personally been attacked outside the home. Scoring yes to any of these was defined as exposure to community violence. In YC, children’s provision of care to younger children and sick people in the home were measured using a binary checklist adapted from the Multidimensional Assessment of Caring Activities questionnaire [20] based on qualitative research and piloting with South African children in the sampled communities. Caring for younger children in the home included any of: walking the child to school, washing the child, or feeding the child. Caring for sick people in the home included any of: administering medication, dressing, toileting or bathing, helping with mobility, massaging the chest for respiratory relief, or cleaning up bodily fluids. In CCC, positive responses to the binary questions ‘Do you help look after younger kids in your home?’ and ‘Have you ever helped unwell people in your home?’ indicated participants’ provision of care for young children or sick people. In YC, the HIV status of the participant’s caregiver was determined using the youth-report Verbal Autopsy [21], which has shown 89% sensitivity and 93% specificity in South Africa [22]. In CCC, caregivers’ HIV status was determined by self-report. Community-based organisation provision was systematically recorded by standardised logging by CBO directors or managers.

#### Outcomes

Psychological distress was measured using standardised scales validated previously with South African children. In both studies, depressive symptoms were measured using a short-form of the Child Depression Inventory [23] (10 items YC, 9 items CCC, scored 0–2). For both, scores were summed for a total depressive score, with higher scores indicating worse depression (*α* = .67). In both studies, suicidal ideation was determined by a positive response to a binary question about whether participants had thought about killing themselves. Post-traumatic symptoms were measured using different scales in each of YC and CCC. However, as both scales measure the same construct, the total scores were standardised and combined into a single scale. YC used the Child PTSD Checklist [24] which has been validated in South Africa [25] (*α* = .67). CCC used the Trauma Symptom Checklist for Children [26], consisting of ten items scored 0 = never to 3 = almost all the time, with higher scores indicating worse trauma (*α* = .74). Child abuse and domestic conflict and violence were measured using UNICEF items for sub-Saharan Africa and analysed with conservative cut-offs [27]. Physical abuse was measured with two items and was defined as carers using a stick/belt to hit the child, or slapping/punching the child at least weekly; emotional abuse was measured with four items and defined as carers threatening to send the child away, withholding meals, invoking ghosts or harm upon the child, or insulting the child at least weekly. Domestic conflict was measured as adults shouting at each other in the home, while domestic violence was indicated by adults hitting each other in the home, all scored 0 = never to 3 = weekly. In YC, occurrences of domestic conflict and violence were measured in the past week, whereas CCC participants indicated whether domestic conflict and violence occurred weekly in their home. A positive response on either set of items was subsequently defined as weekly domestic conflict or violence for the total sample. Household praise was measured as whether the child received praise for behaving or doing something well. The response values between CCC and YC were slightly different and consequently defined as: regular praise (YC: always or often, CCC: often) or irregular praise (YC: sometimes, almost never, or never; CCC: rarely or never). Finally, the HIV status of the child and access to HIV testing was measured through caregiver self-report. In YC, these items were only measured at follow-up. However, due to the young age and sexual inactivity of the current sample, HIV transmission was assumed to be largely vertical. Therefore, child HIV status at follow-up was considered to be a proxy for baseline and used as such in the current analyses. An aggregate measure of cumulative deprivation (scored between 0 and 7) was computed by adding up the presence of each of the following factors: at least one parent being deceased, being HIV-positive, having an HIV-positive caregiver, caring for other children, caring for sick people, having seen someone being attacked, and living in an overcrowded household. The higher the score, the more of these factors are present and thus the higher the child’s cumulative deprivation.

#### Analyses

A three-step analysis strategy was carried out using IBM SPSS 21.0 on the combined sample of children receiving and not receiving CBO support. First, differences between participants lost to and retained at follow-up were tested using chi-square (for categorical variables) and t-tests (for continuous variables). Second, in order to determine the simple differences between children attending CBOs and those not attending CBOs, the total sample and YC/CCC sub-samples were described on all variables at follow-up and differences between participants with and without CBO contact were tested using chi-square (for categorical variables) and t-tests (for continuous variables). Third, to test the effects of CBO contact on psychosocial outcomes over time, separate multiple logistic (for binary outcomes) and linear (for continuous outcomes) regression analyses were conducted for each outcome at follow-up, with CBO contact as the independent variable and controlling for gender, age and cumulative deprivation. Except for stigma, peer problems, conduct problems, and prosocial behaviour, which were not measured at baseline, all analyses examined the effect of CBOs on outcomes at follow-up control for these same outcomes at baseline.

## Results

### Community-based organisation provision

Twenty-four CBOs participated in the study. Directors or project managers provided data on composition, funding and delivery of services. CBOs were set up between 1994 and 2012 mostly by people from the community (n = 17), with 7 set up by an external organisation. Inspiration for establishing an NGO was local for 20 and international for 4. Of the 24 CBOs, 12 were located in a village, 8 in a small town, and 4 in a large city. More than half of CBOs were funded from multiple sources (n = 14). Most CBOs (n = 20) employed both paid staff and volunteers, numbering between 5 and 86 (mean = 20, SD = 11, median = 13), and the number of volunteers ranged between 1 and 51 (mean = 11, SD = 14, median = 7). Four CBOs were administered by volunteers only. Most staff and volunteers had a tertiary degree qualification or a certificate and worked full time. The local community played a major role in helping to establish and maintain most CBOs, either by providing support, raising funds or offering contributions such as food, premises or volunteers. Yet, 2 CBOs reported that the local community made things difficult for the group. The number of children enrolled ranged between 35 and 3060 (mean = 578, SD = 787, median = 278). All CBOs provided services for children and adolescents, but fewer (n = 16) provided services for toddlers (2 years and under). Services provided were primarily aimed at vulnerable people, including children and families affected by HIV. Children came from local or neighbouring villages, towns or cities. Most CBOs had to go out and seek those who needed help (41.7%), 27.1% had children and families coming directly to the organisation, and 31.3% had someone referring the child or family. 42.4% of CBOs received referrals from schools, 36.4% received referrals from social workers, police or pastors, and 21.2% received referrals from clinics or hospitals. The majority of CBOs were full (n = 21), and only 3 had capacity left to take in more children. Almost half reported that they visited children every day (n = 11), 6 had weekly visits and 7 had monthly visits. Most CBOs had visits lasting one hour or less (n = 13), 9 had visits lasting 2 to 5 hours, and 2 had visits lasting all day. Most CBOs (n = 19) saw children at their home, 17 CBOs also saw children at the organisation’s premises, and 13 CBOs also visited children at their school. Services provided included: social grants or direct income support, food/nutrition, psychosocial and emotional support, home-based care, educational support, play supervision, early childhood development support, skills building and training, medical provision or emergency support. Provisions such as food support and assistance with accessing social grants were widely available, whereas skills building and training services were less frequently provided. Fewer CBOs provided medical services (e.g., supplies, emergency), or income support directly to children and families, yet they assisted those in need to access these services, for example through referrals. In addition, the majority of CBOs (n = 21) supported learners to access schooling, and 3 CBOs assisted with school fees. 23 CBOs had contact with children’s caregivers and discussed the child’s progress with the caregiver, and only 1 reported never having contact. CBOs with caregiver contact had visits every month (n = 16), weekly (n = 5) or daily (n =). CBOs also provided carers with other types of input, such as health education and information, help with accessing other services, skills building and training, and health services.

### Differences between participants lost to and retained at follow-up

Follow-up rates were high (86.5% in the CCC study and 97.5% in the YC study). Despite this an analysis was carried out of the differences observed at baseline between participants retained at and lost-to-follow-up (regardless of CBO contact). These are summarised in Table 1, including t- and p-values for each comparison. Notably, participants lost-to-follow-up were largely similar on most variables compared to those retained, including on: gender, school enrolment, school grade, informal housing, age of primary caregiver, household employment, HIV status of caregiver, physical abuse, caregiver praise, having been attacked outside the home, depressive symptoms, suicidal thoughts, responsibility for sick people, and domestic violence. However, children who were lost to follow-up tended to be younger than those who were retained (M = 11.21, SD = 1.2 and M = 11.47, SD = 1.2, respectively). Moreover, children who were retained at follow-up had primary caregivers who were their biological parents more often than those lost to follow-up (67.7% versus 52.8%). This is further reflected in the finding that more participants lost to follow-up were orphans (n = 44, 41.5%) compared to those retained at follow-up (n = 520, 30.0%). Violence and abuse rates were significantly lower among children lost to follow-up compared to those retained: fewer had experienced regular emotional abuse (2.8% versus 8.0%), experienced domestic conflict (15.9% versus 25.5%), or seen someone be attacked on the street (25.2% versus 36.3%). In contrast, more children who were lost-to-follow-up were responsible for caring for younger children (36.4%) compared to those retained at follow-up (25.2%). Although the one-year follow-up retention rate was extremely high, participants who were deceased or untraceable were in some ways more vulnerable and thus findings may slightly under-estimate risks.

**Table 1:** Baseline comparison between participants who were lost to and retained at follow-up.

	Total, N = 1848	Lost to follow-up			Retained at follow-up			χ^2^ or *t* (p-value)
		*Total*, *N = 107*	*CCC*, *N = 63*	*YC*, *N = 44*	*Total*, *N = 1741*	*CCC*, *N = 383*	*YC*, *N = 1358*	
**Female gender**	1011 (54.7%)	54 (50.5%)	28 (44.4%)	26 (59.1%)	957 (55.0%)	197 (51.4%)	760 (56.0%)	0.824 (.364)
**Age**	**11.5 (1.2)**	**11.21 (1.2)**	10.89 (1.3)	11.68 (1.1)	**11.47 (1.2)**	10.95 (1.3)	11.62 (1.1)	**2.137 (.033)**
**School non-enrolment**	12 (0.6%)	1 (0.9%)	0 (0.0%)	1 (2.3%)	11 (0.6%)	3 (0.8%)	8 (0.6%)	0.143 (.705)
**Grade**	5.3 (1.5)	5.21 (1.6)	4.97 (1.6)	5.55 (1.6)	5.33 (1.5)	5.03 (1.5)	5.42 (1.5)	0.856 (.392)
**Informal housing**	485 (26.2%)	32 (29.9%)	14 (22.2%)*	18 (40.9%)	453 (26.0%)	46 (12.0%)*	407 (30.0%)	0.787 (.375)
**Age of primary carer**	42.3 (12.5)	41.96 (15.4)	41.30 (16.4)*	42.91 (13.79)	42.30 (12.3)	46.52 (15.1)*	41.11 (11.0)	0.224 (.823)
**Biological parent is primary carer**	**1234 (66.8%)**	**57 (52.8%)**	27 (42.9%)	30 (68.2%)	**1177 (67.7%)**	159 (41.5%)	1018 (75.0%)	**10.187 (.001)**
**≥1 Employed person in the household**	1318 (71.4%)	68 (63.6%)	36 (57.1%)	32 (72.7%)	1250 (71.8%)	213 (55.6%)	1037 (76.4%)	3.388 (.066)
**Orphan (at least one parent died)**	**564 (30.7%)**	**44 (41.5%)**	34 (54.8%)	10 (22.7%)	**520 (30.0%)**	235 (62.5%)	285 (21.0%)	**6.238 (.013)**
**Close person died recently**	**986 (53.4%)**	**47 (43.9%)**	19 (30.2%)	28 (63.6%)	**939 (53.9%)**	95 (24.8%)	844 (62.2%)	**4.058 (.044)**
**HIV-positive carer**	379 (20.5%)	19 (17.8%)	10 (15.9%)	9 (20.5%)	360 (20.7%)	57 (14.9%)	303 (22.3%)	0.527 (.468)
**Weekly emotional abuse**	**142 (7.7%)**	**3 (2.8%)**	0 (0.0%)	3 (6.8%)	**139 (8.0%)**	10 (2.6%)	129 (9.5%)	**3.813 (.051)**
**Weekly physical abuse**	97 (5.2%)	5 (4.7%)	1 (1.6%)	4 (9.1%))	92 (5.3%)	1 (0.3%)	91 (6.7%)	0.076 (.783)
**Regular praise from carer**	1289 (69.8%)	83 (77.6%)	57 (90.5%)*	26 (59.1%)	1206 (69.3%)	298 (77.8%)*	908 (66.9%)	3.291 (.070)
**Attacked outside home**	162 (8.8%)	13 (12.1%)	10 (15.9%)	3 (6.9%)	149 (8.6%)	43 (11.3%)	106 (7.8%)	1.620 (.203)
**Seen someone be attacked**	**658 (35.6%)**	**27 (25.2%)**	27 (42.9%)	0 (0.0%)***	**631 (36.3%)**	163 (42.7%)	468 (34.5%)***	**5.348 (.021)**
**Depressive symptoms**	1.10 (1.8)	1.41 (2.1)	1.19 (1.8)	1.73 (2.4)	1.09 (1.8)	0.77 (1.2)	1.17 (1.9)	1.832 (.067)
**Suicidal thoughts**	71 (3.8%)	3 (2.8%)	0 (0.0%)	3 (6.8%)	68 (3.9%)	14 (3.6%)	54 (4.0%)	0.331 (.565)
**Care for younger children**	**477 (25.9%)**	**39 (36.4%)**	32 (50.8%)	7 (15.9%)	**438 (25.2%)**	179 (46.7%)	259 (19.1%)	**6.651 (.010)**
**Care for sick people**	537 (29.1%)	35 (32.7%)	24 (38.1%)	11 (25.0%)	502 (28.9%)	144 (37.6%)	358 (26.4%)	0.715 (.398)
**Weekly domestic conflict**	**460 (24.9%)**	**17 (15.9%)**	3 (4.8%)	14 (31.8%)	**443 (25.5%)**	20 (5.2%)	423 (31.3%)	**4.938 (.026)**
**Weekly domestic violence**	79 (4.3%)	5 (4.7%)	2 (3.2%)**	3 (6.8%)	74 (4.3%)	1 (0.3%)**	73 (5.4%)	0.043 (.835)

*Note*. Data are mean (SD) or N (%). Difference statistic is chi-square for categorical variables and t-score for continuous variables. The difference statistic shows the difference between total retained for follow-up and total lost to follow-up per variable across both studies; statistically significant differences are bolded. Asterisks denote differences between retained at and lost to follow-up separately for both studies (YC and CCC).

* p < .05

** p < .01

*** p < .001.

### Socio-demographic differences between groups at follow-up

As shown in Table 2, at follow-up children who attended CBOs significantly differed from those who did not on several socio-demographic variables. Fewer children who attended CBOs lived in informal housing compared to those who did not attend CBOs (14.4% versus 21.4%). In contrast, fewer CBO-attending children lived in a household where at least one person was employed (59.0% versus 76.4%), and double orphan rates were much higher among children attending CBOs (25.7% versus 2.1%). Additionally, caregivers of children attending CBOs were more often HIV-positive (17.0% versus 11.3%; %). Participants in contact with CBOs were also more likely to report caring for younger children (73.1% versus 14.7%) and sick people (65.2% versus 14.8%). Exposure to community violence was also greater amongst children with CBO contact compared to those without: more children attending CBOs had seen someone attacked on the street (42.2%) as compared to those who did not attend CBOs (33.4%). Families of children attending a CBO were more often in receipt of any grant (88.3%) than families without CBO contact (79.1%). No differences were observed between participants with and without CBO contact on school enrolment, being attacked outside the home, or receipt of grants specifically meant for child support.

**Table 2:** Differences on socio-demographic variables between participants with and without CBO contact at follow-up.

	With CBO contactN = 446	Without CBO contact N = 1402	χ^2^ (p-value)
**Informal housing**	**54 (14.4%)**	**250 (21.4%)**	**9.00 (.003)**
**School non-enrolment**	7 (1.9%)	22 (1.6%)	0.13 (.715)
**≥1 Employed person in the household**	**222 (59.0%)**	**1037 (76.4%)**	**44.74 (< .001)**
**Mother died**	**65 (17.6%)**	**73 (5.4%)**	**58.74 (< .001)**
**Father died**	**87 (23.5%)**	**191 (14.1%)**	**19.18 (< .001)**
**Double orphan**	**97 (25.7%)**	**30 (2.1%)**	**248.61 (< .001)**
**HIV-positive carer**	**65 (17.0%)**	**154 (11.3%)**	**8.58 (.003)**
**Attacked outside home**	34 (9.2%)	123 (9.1%)	0.00 (.952)
**Seen someone be attacked**	**160 (42.2%)**	**468 (33.4%)**	**10.20 (.001)**
**Care for younger children**	**211 (47.3%)**	**199 (14.7%)**	**505.72 (< .001)**
**Care for sick people**	**169 (37.7%)**	**200 (14.8%)**	**389.93 (< .001)**
**Receipt of child support or foster child grant**	304 (79.4%)	1047 (77.2%)	0.81 (.370)
**Receipt of any grant**	**338 (88.3%)**	**1072 (79.1%)**	**16.46 (< .001)**

*Note*. Data are N (%) and difference statistic is chi-square. Statistically significant differences are bolded.

### Is CBO contact at baseline associated with more positive psychosocial outcomes at follow-up?

Tables 3 and 4 summarise the logistic (for binary outcomes) and linear (for continuous outcomes) regressions analysing the association between CBO contact and psychosocial outcomes, controlling for the relevant outcome at baseline (where possible), gender, age, and cumulative deprivation. It was found that participants attending CBOs had lower odds of experiencing weekly domestic conflict between adults in their home (OR 0.17; 95% CI 0.09, 0.32), domestic violence (OR 0.22; 95% CI 0.08, 0.62), or abuse (OR 0.11; 95% CI 0.05, 0.25) at follow-up compared to participants without CBO contact. Likewise, attending a CBO was associated with lower odds of suicidal ideation (OR 0.41; 95% CI 0.18, 0.91), fewer depressive symptoms (B = -0.40; 95% CI -0.62, -0.17), less perceived stigma (B = -0.37; 95% CI -0.57, -0.18), fewer peer problems (B = -1.08; 95% CI -1.29, -0.86) and fewer conduct problems (B = -0.77; 95% CI -0.95, -0.60) at follow-up. Finally, CBO contact was associated with indicating more prosocial behaviours at follow-up (B = 1.40; 95% CI 1.13, 1.67). No associations were observed between CBO contact and parental praise or post-traumatic symptoms.

**Table 3:** Longitudinal associations between CBO contact and binary outcomes.

Outcome variable	Odds Ratio (95% CI)	p-value
**Any weekly abuse (physical or emotional)**	**0.112 (0.051–0.247)**	**< .001**
**Regular parental praise**	1.062 (0.802–1.406)	.676
**Suicidal ideation**	**0.408 (0.183–0.909)**	**.028**
**Weekly domestic conflict**	**0.171 (0.090–0.324)**	**< .001**
**Weekly domestic violence**	**0.218 (0.077–0.622)**	**.004**

*Note*. Analyses are multiple logistic regression analyses, conducted separately for each outcome variable at follow-up. For all analyses, the predictor variable was CBO contact and the covariates were gender, age, cumulative deprivation, and the outcome variable at baseline. Cumulative deprivation was a summed score of the following: orphanhood, HIV-positive carer, HIV-positive child, child cares for other children, child cares for sick people, child has seen someone being attacked, and child lives in an overcrowded household. Statistically significant differences are bolded.

**Table 4:** Longitudinal associations between CBO contact and continuous outcomes.

Outcome variable	B (unstandardized) coefficient (95% CI)	p-value
**Depressive symptoms**	**-0.395 (-0.621, -0.169)**	**.022**
**Post-traumatic symptoms (standardized)**	-0.010 (-0.135, 0.114)	.874
**Stigma**^a^	**-0.372 (-0.568, -0.175)**	**< .001**
**Peer problems**^a^	**-1.076 (-1.288, -0.864)**	**< .001**
**Prosocial behaviours**^a^	**1.402 (1.130, 1.674)**	**< .001**
**Conduct problems**^a^	**-0.773 (-0.951, -0.595)**	**< .001**

*Note*. Analyses are multiple linear regression analyses, conducted separately for each outcome variable at follow-up. For all analyses, the predictor variable was CBO contact and the covariates were gender, age, cumulative deprivation, and the outcome variable at baseline, except where otherwise noted. Cumulative deprivation was a summed score of the following: orphanhood, HIV-positive carer, HIV-positive child, child cares for other children, child cares for sick people, child has seen someone being attacked, and child lives in an overcrowded household. Statistically significant differences are bolded.

^a^ These analyses did not control for the outcome variable at Time 1, as these variables were only measured in both studies at follow-up.

## Discussion

This study provides longitudinal outcome data for children in South Africa with CBO contact compared to those with no CBO contact. Initially it is important to note that children who were lost to follow-up may have differed somewhat to those who were retained, despite the high retention rates. This needs to be taken into account when considering the extent to which these findings are generalizable.

Participants attending CBOs had lower odds of experiencing weekly domestic conflict, domestic violence, or abuse at follow-up compared to participants without CBO contact. Given the longitudinal nature of the data it does appear that CBO attendance is associated with reduced violence and abuse experiences for children. This can be accounted for by a number of possible pathways. Parents who are less likely to use harsh discipline may also be more likely to attend CBOs resulting in a potential selection bias. Attendance at the CBO may be associated with provisions that alleviate some of the dire poverty experiences and in turn diffuse family tension and anxiety. This is cautiously supported by the fact that in this study it was found that families with CBO contact were more often in receipt of grants than families without CBO contact. However, such indirect pathways would need to be studied in detail to ascertain direct causal links. In addition, many CBO interventions, such as parenting classes, home visiting, referral for support and CBO resources may have directly affected the outcomes for children. The data seems to be clearly indicating that CBO attendance has advantages for children in terms of exposure to domestic conflict and violence and abuse in terms of harsh punishment and family actions.

In terms of mental health and behaviour, CBO exposure was associated with lower odds of suicidal ideation, fewer depressive symptoms, less perceived stigma, fewer peer problems and fewer conduct problems at follow-up. In addition, CBO contact was associated with more prosocial behaviour at follow-up. These results suggest that CBO exposure is associated with behavioural and mental health benefits over time. However, exposure to CBO was not associated with any differences in post-traumatic symptoms. It thus seems that the CBO may be proficient in providing support, input on behaviour and even affect mood, but trauma is more difficult to have an impact on. It may very well be that more specialised help is necessary to alleviate more severe psychopathological problems such as trauma. Overall, the mechanisms behind the positive effect of CBO contact on mental health and behaviour are still unclear. Although many CBO interventions may be mediated through parental training and support [16], we found no associations between CBO contact and parental praise. The literature shows that positive parenting is more complex than praise alone, with abuse having negative effects and boundaries with strong parental supervision having positive effects [28].

The limitations of this study are the use of two different data sources and the lack of random allocation to groups. The studies employed different methods of sampling with different data collection procedures and team members. They also took place at slightly different times, although over the same period. Some measures used are also slightly different, which may have impacted the results. However, given the nature of CBO provision and the fact that most CBOs are already set up and operational, there is real value in using pragmatic and quasi-experimental approaches to compare CBO-attendees to random samples in the population [29]. The overlap of age bands for the two studies restricted the analysis to 9–13 year old children and thus the findings for older or younger attenders cannot be inferred. Despite these limitations the data clearly shows that 9–13 year old children exposed to CBO services show scores associated with marked benefit both in terms of their violence and abuse experiences as well as an array of mental health and behavioural outcomes. The longitudinal data allows for control of baseline levels for most outcomes and still shows associated benefits. Future research could valuably examine by what mechanisms CBOs impact child outcomes over time.

With increasing resource challenges, it is essential to identify whether community-based provision is associated with measurable benefits for children and families. And indeed, these data show specific benefits for hard-to-reach and challenged families in the HIV/AIDS era. For many CBOs the provision is based on volunteer providers, scant resources and limited premises and providers. Despite this, their proximity in the community, their ongoing and sustained relationship with families and the experiences and support provided seem to be worthy of investment. It is well-established that problems in childhood have far-reaching effects and that there is a good investment case for interventions for children to interrupt the cycle of abuse, violence, poor mental health and risk behaviours. This data suggests that CBO provision may well be a positive resource in the armoury.
